# Quality of life and mental health of adolescents: Relationships with social media addiction, Fear of Missing out, and stress associated with neglect and negative reactions by online peers

**DOI:** 10.1371/journal.pone.0286766

**Published:** 2023-06-07

**Authors:** Vu Anh Trong Dam, Nam Gia Dao, Duy Cao Nguyen, Thuc Minh Thi Vu, Laurent Boyer, Pascal Auquier, Guillaume Fond, Roger C. M. Ho, Cyrus S. H. Ho, Melvyn W. B. Zhang

**Affiliations:** 1 Institute for Global Health Innovations, Duy Tan University, Da Nang, Vietnam; 2 Faculty of Medicine, Duy Tan University, Da Nang, Vietnam; 3 Institute of Health Economics and Technology, Hanoi, Vietnam; 4 Research Centre on Health Services and Quality of Life, Aix Marseille University, Marseille, France; 5 Department of Psychological Medicine, Yong Loo Lin School of Medicine, National University of Singapore, Singapore, Singapore; 6 Institute for Health Innovation and Technology (iHealthtech), National University of Singapore, Singapore, Singapore; 7 Lee Kong Chian School of Medicine, Nanyang Technological University Singapore, Singapore, Singapore; Sabzevar University of Medical Sciences, ISLAMIC REPUBLIC OF IRAN

## Abstract

Social networking is proliferating globally and in Vietnam, but this led to several negative aspects of adolescents’ health, including physical activity, sleep quality, and depressive and anxiety symptoms. This study aimed to identify the relationship between social media usage and examine risk factors (Fear of Missing out and Risk of Neglect) on social media usage, and the overall quality of life and mental health among individuals actively using social media networks. An online cross-sectional study was conducted in three cities in Vietnam (Hanoi, Tuyen Quang, and Can Tho) from September through to October 2021. A structured questionnaire assessed for characteristics of social media use and other associated factors. 1891 participants were recruited, with 98.4% having access to social media. s. Factors like “PHQ-9 score”, “Problematic Internet use”, and “Time average used social media per day”, were negatively associated with the EQ5D5L Index. By contrast, “Gender”, and “Using smartphone” were positive factors of the EQ5D5L Index. “FOMO score” and “self-harm and suicide” were positive factors of the PHQ-9 score while “Using smartphone” was negative. In terms of self-harm and suicide, “FOMO score” and “Problematic Internet use” were positive factors, by contrast, “Using smartphone” was a negative factor. This is the first study to examine social media addiction among Vietnamese adolescents, its relationship with FOMO score, stresses associated with rejection and neglect, and the overall quality of life. Our results highlighted there is a relationship between FOMO score and impaired overall quality of life, increased depressive symptoms, and an association between stresses relating to negative rejection and FOMO score.

## Introduction

According to recent statistics, an estimated 58.4% of the global population uses social media [1]. The average amount of time individuals spend using social media daily was 2 hours and 27 minutes [1]. One of the contributory reasons for the popularity of social media usage might be the advances in information and communication technologies and the overall increased penetration rates of smartphone devices globally. In fact, in a low and middle-income country like Vietnam, the smartphone penetration rate is estimated to be 73.5%, with a projected growth to that of 85% by the end of 2022 [2]. Other reasons that account for the increase in the use of social media among individuals are how these social networks allow not only for the ease of communication but how they enable individuals to link up and form a virtual relationship that is not confined by any geographical barriers [3]. Social media also allows for information sharing [4]. Yoon S et al. (2021) have previously highlighted how social media has been tapped upon to actively engage patients and the general public. While the use of social media has clearly multiple benefits, in recent years, there have been increasing concerns relating to the excessive use of social media. Prior studies have examined the impact of one’s addiction to social networks like Facebook and its consequential impact on one’s psychosocial functioning. For example, Busalim et al. (2019) reported how self-esteem has hurt Facebook addiction for students who were not addicted; and how Facebook addiction led to worsening academic performance [5]. In recent years, with the increasing popularity of other social media networks such as Instagram, they have also been investigated. Souza et al. examined the relationship between depression and Instagram addiction among 131 students in India, aged between 12–23 years old. They reported there is an association between the severity of one’s addiction to Instagram and one’s depressive scores [6]. More recently, studies have explored not just the prevalence of social media addiction, but how the use of different platforms contributes to varying levels of addiction [7]. It was found that individuals who used more visual forms of social media, such as Instagram and Tik Tok, had higher levels of social media addiction as compared to conventional platforms like Whatsapp and Tik Tok [7].

While some studies and questionnaires examined the severity of one’s addiction to different social media platforms, there remains to date no formal diagnostic criteria for social media addiction, unlike that of Internet Gaming Disorder, which was recently included in the ICD-11 classification system [8]. Some researchers have social media addiction by defining it as a form of behavioral addiction, in which an individual has compulsive engagement with social media networks, to the extent that it has a clear consequential impact on other areas of functioning [9]. To ascertain if an individual has a social media addiction, they need to fulfill the following symptoms, namely that of (a) salience of behavior, (b) tolerance, (c) usage of social media for mood modification, (d) relapse, (e) withdrawal symptoms when not using and (f) impact on one’s functioning [9]. Some researchers have advocated for the concept of social media addiction, given that it shares not only some of the core addiction clinical manifestations but also shares some common risk factors for substance and other behavioral addictions, such as young age of onset, common risk factors such as impulsivity [10,11]. However, social media addiction remains a concept that is not universally recognized nor accepted, as some researchers argue that labeling one with social media addiction is an overpathologisation of excessive use of ordinary social media [10,11]. Some of the arguments against social media addiction are that the research into this area remains exploratory at present [12]. It remains hard to differentiate what constitutes social media addiction versus one who is over-using it, and this differentiation remains highly debatable [13].

Due to the absence of a formal classification and diagnostic criteria for social media addiction, and the existence of numerous questionnaires, the prevalence of social media addiction has been reported to be rather a heterogenous [9], ranging from between 1 to 15% in the United Kingdom and 0–11% in the United States. While there have been statistics about the penetration and adoption of smartphone devices in Vietnam, no prior study has specifically examined social media addiction in Vietnam. However, there have been prior studies examining the impact of specific social media networks. For example, Zhang et al. [14] has previously investigated Vietnamese youths’ perception of health-related information on Facebook, and more recently, Diep et al. [15] has examined health science students’ usage of social media for educational purposes in Vietnam.

Spending excessive time and being addicted to social media is just one of the consequences of excessive social media use. Studies have reported that social media use has impacted not only one’s physical activity and sleep quality [11], but also university students’ academic performance [16]. Other studies, such as the review conducted by Karim et al. [17], has reported how social media users have had an impact on both depressive and anxiety symptoms, and that the risk factors for these symptoms were that of the amount of time one spent on the Internet, their activity and one’s addiction to social media. Apart from evidence highlighting the association between excessive social media use/addiction and psychiatric disorders, other studies have explored factors that might predispose individuals toward social media use and addiction. One of the factors is that of the Fear of Missing out (FOMO) [18]. Due to FOMO, individuals face constant preoccupation with needing to use social media, for they worry about missed opportunities when they are offline, or not being able to connect with others [18]. The theory underlying the experience of FOMO is that of the self-determination theory, whereby individuals have a psychological need to connect with others. Doing so helps with their emotional self-regulation and psychological well-being [19]. Thus, individuals who experience heightened levels of FOMO are more likely to use social media to help them with these psychological needs. Studies such as Bloemen et al. have highlighted how FOMO could predict Internet addiction and reported there being a strong reciprocal relationship between FOMO and social media usage [20]. There have also been studies that have reported the impact of FOMO on individuals, in that FOMO might result in sleep disturbances, experiences of emotional tension, anxiety, and affects overall well-being [18,21,22]. Another factor that might result in individuals using more social media is the stress of not receiving positive comments or likes by peers or others online [23]. In fact, the fear of not receiving negative comments could be a risk factor that predisposes individuals to spend more time online to try and maintain positive relationships with others [23].

The conduct of this study is particularly timely. As evident from the literature review, a lack of research has been undertaken in Vietnam, examining social media addiction and its related risk factors. To date, no research has been undertaken to examine the impact of FOMO and the stresses associated with neglect and negative reactions to one’s social media usage. The examination of this topic is important, given the increasing penetration rates of smartphone ownership in Vietnam, and the increasing use of social media networks in the country. Unlike the situation in Vietnam, other similar studies have been conducted overseas, exploring social media and its associated factors. Gori et al. (2023) sampled a total of 470 social media users and reported that preoccupied and fearful attachment patterns were associated with problematic social media use [24]; and these styles of attachments were in turn related to FOMO and self-esteem [24]. Other studies, such as that by Moretta et al. (2023) have demonstrated the association between COVID-19 pandemic-related stress and social media use [25]. Given this, this study aims to determine the extent of social media usage, examine risk factors (FOMO and Risk of Neglect) on social media usage, and the overall quality of life and mental health among individuals who were actively using social media networks. The research question was to determine the rates of social media and the associated factors associated with excessive social media use.

## Materials and methods

### Study design and participants

To achieve the aims of this study, an online cross-sectional study was conducted, and participants were recruited from three cities in Vietnam: Hanoi, Tuyen Quang, and Can Tho. The recruitment lasted from September through to October 2021. Participants were included in the study if they were (a) currently residing in Vietnam, (b) were willing to participate in the online study, and capable of providing consent online, and (c) had both the physical and psychological capacity to answer the questionnaires. Participants were excluded if they had any underlying cognitive impairments.

### Sample size and sampling technique

We used the snowball sampling approach through a four-step process, which entailed starting with student union presidents of schools as core groups and forwarding them a website link containing details about the study and survey. They were then requested to share the website link with others in their network through email, social media, or other online platforms. The people invited continued to take the survey and encouraged others to do so.

To estimate the sample size that was required for this study, the formula to estimate the mean score of EQ5D5L among participants was utilized. With an expected standard deviation of the EQ5D5L score of 0.9 [26], ad marginal error of 0.2, and a type I error rate of 5%, at least 1898 participants were needed. At the end of the data collection duration, a total of 1929 individuals were invited for the study and 1891 participants completed the questionnaire. Hence, the completion rate was 98.03%.

### Measure and instruments

A structured questionnaire was utilized and it includes questions that sampled the following domains: 1) Socio-demographic information, 2) Characteristics of social media use and whether one has problematic internet use, 3) One’s experiences relating to Fear of Missing out, 4) Stresses associated with Neglect and Negative Reactions by online peers and lastly, one’s 5) quality of life and mental health. This structured questionnaire was uploaded and hosted on an online survey platform, that of SurveyMonkey (Surveymonkey.com). We have decided to use this approach, as it was not only economical but saves time with regards to the dissemination of the questionnaire. More importantly, the web-based platform was intuitive in design and familiar to adolescents. The use of this method also allowed us to reach a large sample of adolescents rapidly [27]. The survey was originally piloted with 20 students of different ages, genders, and parents’ characteristics to ascertain the Vietnamese-translated questionnaires’ validity. Data collection was commenced following the successful validation of the online survey system. The validation helped ensure that no technical issues might affect the administration and collation of data.

The structured questionnaire comprised of the following domains:

#### Outcome variables

*The Patient Health Questionnaire (PHQ-9) (9 items)* was used to measure the severity of depressive symptoms experienced in the last 2 weeks. This scale measures cognitive/affective and somatic disorders to screen for depression and suicide risk. Answers were rated on a 4-point Likert scale (from 0 “not at all” “to 3 “nearly every day”). The total scores ranged from 0 to 27. Depression severity was characterized as none (0–4); mild (5–9), moderate (10–14), moderately severe (15–19), and severe (≥20). A PHQ9 ≥ 10 was the cut-off point for major depression with a sensitivity of 88% and a specificity of 88% [28].

*EuroQol-5 Dimensions-5 Levels (EQ-5D-5L)* were used to indicate the quality of life of participants. There were five domains including Mobility, Self-care, Usual Activities, Pain/Discomfort, and Anxiety/Depression. Each question had a score from 1 “Extreme problems” to 5 “No problem”. In this study, we used the Vietnamese version that has been validated and in use, with scores ranging from -0.5115 to 1 [29]. Participants with higher scores indicated the higher quality of life. The Cronbach’s alpha was excellent at 0.91.

#### Covariate

*Socioeconomic status*. Respondents were asked about and reported their socio-demographic information. Questions asked included age, gender (man/woman), location of residence (urban areas, rural/mountainous areas), and occupation (students, others).

*Social media characteristics*. To ascertain the nature of social media use, we asked two questions. Firstly, respondents were asked if they used smartphones to assess social media (Yes/No). Secondly, respondents were asked to quantify the amount of time they spend on social media daily (less than 2 hours, between 3–5 hours, 6–8 hours, and more than 8 hours).

*Fear of Missing Out Scale (FOMO)*. We use the FOMO Scale to measure the degree to which one fears missing out on social events, especially events involving friends and the frequency one uses social media to stay (hyper) connected [30]. There were 10 unidimensional scales, with questions that required 5-point Likert-type responses. The total scores ranged from 10 to 50, in which higher scores indicated higher levels of Fear of Missing Out [30]. The Cronbach’s alpha was good at 0.88.

*Stress associated with neglect and negative reactions by online peer*. We used this scale to assess two aspects of users’ experiences on social media. There were a total of 8 items, which corresponded with 2 aspects that were associated with experiences of neglect by other users (4 items) and negative reactions by other users (4 items) [31]. Each item was rated on a 5-point Likert scale ranging from 1 “completely disagree” to 5 “completely agree”. Participants who endorsed higher scores experienced higher levels of experience with being neglected by other users [31]. The Cronbach’s alphas of the two domains were excellent at 0.97 and 0.91, respectively.

*Problematic Internet use*. The Problematic Internet Use Questionnaire SF-6 (PIUQ SF-6) was used to evaluate respondents’ Internet use patterns. This questionnaire comprised 6 items, each item was scored from 1 “Never” to 5 “Always”. The total scores ranged from 6 to 30, a higher total score indicated increased problematic internet use [32]. The Cronbach’s alpha was good at 0.83.

### Data analysis

We used STATA version 16 to analyze the data. The Listwise Deletion method was conducted to eliminate missing data. We described continuous variables by the mean and standard deviation (SD), meanwhile, categorical variables were described as frequencies and percentages. The difference between gender groups was analyzed using Chi-squared and Kruskal-Wallis tests.

A set of covariates, including individual characteristics, social media characteristics, and problematic internet use were used in the models. Multivariate regression was used to identify the factors related to EQ5D Index and multivariate Tobit regression to gauge the factors relating to the PHQ-9 score. Multivariate logistic regression was employed for identifying factors linked to self-harm and suicide. The stepwise forward method was implemented to produce reduced models with p < 0.2 as the threshold for included variables. A P-value (P) below 0.05 was regarded as statistically significant. The smartphone use and self-harm/suicide roles were coded as binary variables (0 “No” and 1 “Yes”), while problematic Internet use, average time spent on social media per day, and SS scale were taken as continuous variables.

Using Structural equation modeling, the mediation impacts of fear of missing out on the associations between problematic Internet use, time average used social media per day, using a smartphone, SS scale, EQ5D5L Index, PHQ-9 score, and Self-harm/suicide was determined. The goodness-of-fit indices were examined, including the root-mean-square error of approximation (RMSEA), the comparative fit index (CFI), and the standardized root mean square residual (SRMR), with RMSEA below 0.08, SRMR below 0.08, and CFI higher than 0.09 considered acceptable model fits [33].

### Ethics approval and consent to participate

All procedures performed in studies involving human participants were by the ethical standards of the scientific committee of the Youth Research Institute, Vietnam, and with the 1964 Helsinki Declaration and its later amendments or comparable ethical standards. Participation was completely voluntary, and online informed consent was requested before the study subjects participated in the study. Respondents who were under 16 must have the consent of parents or guardians to participate in this study. Collected data were saved in a secured system and only served the study purposes.

## Results

**Table 1** provided an overview of the individual characteristics of respondents. Of the 1891 participants, 56.7% were women, and the mean age was 17.0 (SD = 2.8). Most were students (92.9%) and are living in urban areas (91.5%). Most used smartphones to access social media (98.4%). 36.4% and 31.1% spend between 3–5 hours and less than 2 hours daily assessing social media. The proportion of man participants who accessed social media for less than 2 hours was higher than woman participants, by contrast, the percentage of women who spent more than 2 hours accessing social media was higher than men (p<0.01).

**Table 1:** Individual characteristics of participants.

Characteristics	Gender						
	Man		Woman		Total		p-value
	n	%	n	%	n	%	
**Total**	818	43.3	1073	56.7	1891	100.0	
**Location**							
Urban areas	748	91.4	982	91.5	1730	91.5	0.95
Rural/mountainous areas	70	8.6	91	8.5	161	8.5	
**Occupation**							
Students	782	95.6	974	90.8	1756	92.9	<0.01
Others	36	4.4	99	9.2	135	7.1	
**Using smartphone to access social media**	802	98.0	1058	98.6	1860	98.4	0.34
**Time average used social media per day**							
< 2 hours	320	40.1	260	24.3	580	31.1	<0.01
3–5 hours	275	34.4	405	37.9	680	36.4	
6–8 hours	121	15.1	257	24.0	378	20.2	
> 8 hours	83	10.4	147	13.8	230	12.3	
	**Mean**	**SD**	**Mean**	**SD**	**Mean**	**SD**	**p-value**
**Age**	16.7	2.3	17.2	3.2	17.0	2.8	0.07
**Fear of Missing Out score (range 10–50)**	22.7	8.3	22.8	7.9	22.7	8.0	0.75
**SS scale**							
Experiences of neglect by other users (range 4–20)	8.2	4.5	8.5	4.2	8.3	4.3	0.04
Negative reactions by other users (range 4–20)	9.0	4.3	9.8	4.1	9.4	4.2	<0.01
**Problematic Internet use (range 6–30)**	9.0	5.1	7.4	3.4	8.1	4.3	<0.01

The mean score of the FOMO questionnaire was 22.7 (SD = 8.0). With the SS scale, the mean score of experiences with being neglected by other users and negative reactions by other users were 8.3 (SD = 4.3) and 9.4 (SD = 4.2), respectively, and women participants had higher scores than man participants (p<0.05). The mean score of problematic internet use was 8.1 (SD = 4.3), in which man participants had a higher PIUQ-SF6 score than woman participants (p<0.01).

**Table 2** described the depression, quality of life, self-harm, and suicide characteristics of participants. More than 30% of respondents have been bothered by self-harm and suicide (20% several days, 6.5% more than half the days, and 4.8% nearly every day). The mean score of EQ-5D-5L and PHQ-9 was 0.80 (SD = 0.31) and 7.5 (SD = 6.2), respectively.

**Table 2:** Depression, quality of life, self-harm, and suicide characteristics of participants.

Characteristics	Gender						
	Man		Woman		Total		p-value
	n	%	n	%	n	%	
**Self-harm and suicide**							
Not at all	556	68.0	743	69.3	1299	68.7	**0.38**
Several days	159	19.4	220	20.5	379	20.0	
More than half the days	57	7.0	66	6.1	123	6.5	
Nearly every day	46	5.6	44	4.1	90	4.8	
	**Mean**	**SD**	**Mean**	**SD**	**Mean**	**SD**	**p-value**
**Quality of Life score (range -0.5515–1)**	0.76	0.36	0.83	0.27	0.80	0.31	0.01
Mobidity (range 1–5)	1.82	1.24	1.52	1.04	1.65	1.14	<0.001
Self-care (range 1–5)	1.60	1.16	1.26	0.83	1.41	1.00	<0.001
Usual Activities (range 1–5)	1.69	1.19	1.41	0.93	1.53	1.06	<0.001
Pain/Discomfort (range 1–5)	1.73	1.17	1.49	0.93	1.60	1.05	<0.001
Anxiety/Depression (range 1–5)	2.00	1.21	1.91	1.10	1.95	1.15	0.45
**Depression (PHQ-9) (range 0–27)**	7.4	6.5	7.6	6.1	7.5	6.2	0.17

**Table 3** revealed that a higher time average used to social media per day was the negative factor that increased the depression score. Meanwhile, participants who had a higher problematic internet use had lower quality of life (Coef = -0.02; 95%CI = -0.02; -0.02) but they tend to have a higher rate of self-harm and suicide (OR = 1.16; 95%CI = 1.13; 1.19). Participants who had a higher FOMO score tended to have a higher quality of life (Coef = 0.004; 95%CI = 0.002; 0.01), Depression score (Coef = 0.23; 95%CI = 0.19; 0.26), and a higher rate of self-harm and suicide (OR = 1.05; 95%CI = 1.01; 1.07). Participants who had a higher negative reaction by other users score had a higher depression score (Coef = 0.10; 95%CI = 0.04; 0.17). Moreover, experiences of neglect by other users were harmful factors that increased the rate of self-harm and suicide (OR = 1.04; 95%CI = 1.01; 1.07). Respondents who intended to self-harm and suicide tended to have a higher depression score (Coef = 7.92; 95%CI = 7.36; 8.47) and lower quality of life score (Coef = -0.05; 95%CI = -0.09; -0.02). Depression score was a negative factor that declined the quality of life of participants (Coef = -0.01; 95%CI = = -0.01; -0.01).

**Table 3:** Multivariate regression to identify factors associated with quality of life, depression, and self-harm and suicide.

Factors	Quality of Life		Depression		Self-harm and suicide	
	Coef.	95%CI	Coef.	95%CI	OR	95%CI
**SOCIO-DEMOGRAPHICS**						
Age (unit: year)					0.96**	0.92; 1.00
Gender (Woman vs Man—Ref)	0.03**	0.00; 0.05			1.28**	1.01; 1.61
Occupation (Others vs Students)			-1.29***	-2.25; -0.34		
**USING INTERNET CHARACTERISTICS**						
Using smartphone to access social media (Yes vs No—Ref)	0.07	-0.03; 0.18			0.48*	0.20; 1.14
Time average used social media per day (vs < 2 hours—Ref)						
3–5 hours			0.61**	0.02; 1.20	0.76**	0.57; 1.00
6–8 hours			1.00***	0.30; 1.69	1.03	0.75; 1.41
> 8 hours			1.68***	0.86; 2.49	1.02	0.71; 1.47
Problematic Internet use (unit: point)	-0.02***	-0.02; -0.02	-0.04	-0.11; 0.02	1.16***	1.13; 1.19
Fear of Missing Out (unit: score)	0.004***	0.002; 0.01	0.23***	0.19; 0.26	1.05***	1.04; 1.07
**SS scale**						
Negative reactions by other users (unit: score)			0.10***	0.04; 0.17		
Experiences of neglect by other users (unit: score)					1.04***	1.01; 1.07
**MENTAL HEALTH**						
Self-harm and suicide (unit: score)	-0.05***	-0.09; -0.02	7.92***	7.36; 8.47		
Depression (unit: score)	-0.01***	-0.01; -0.01				

*** p<0.01

** p<0.05

* p<0.1.

**Fig 1:**
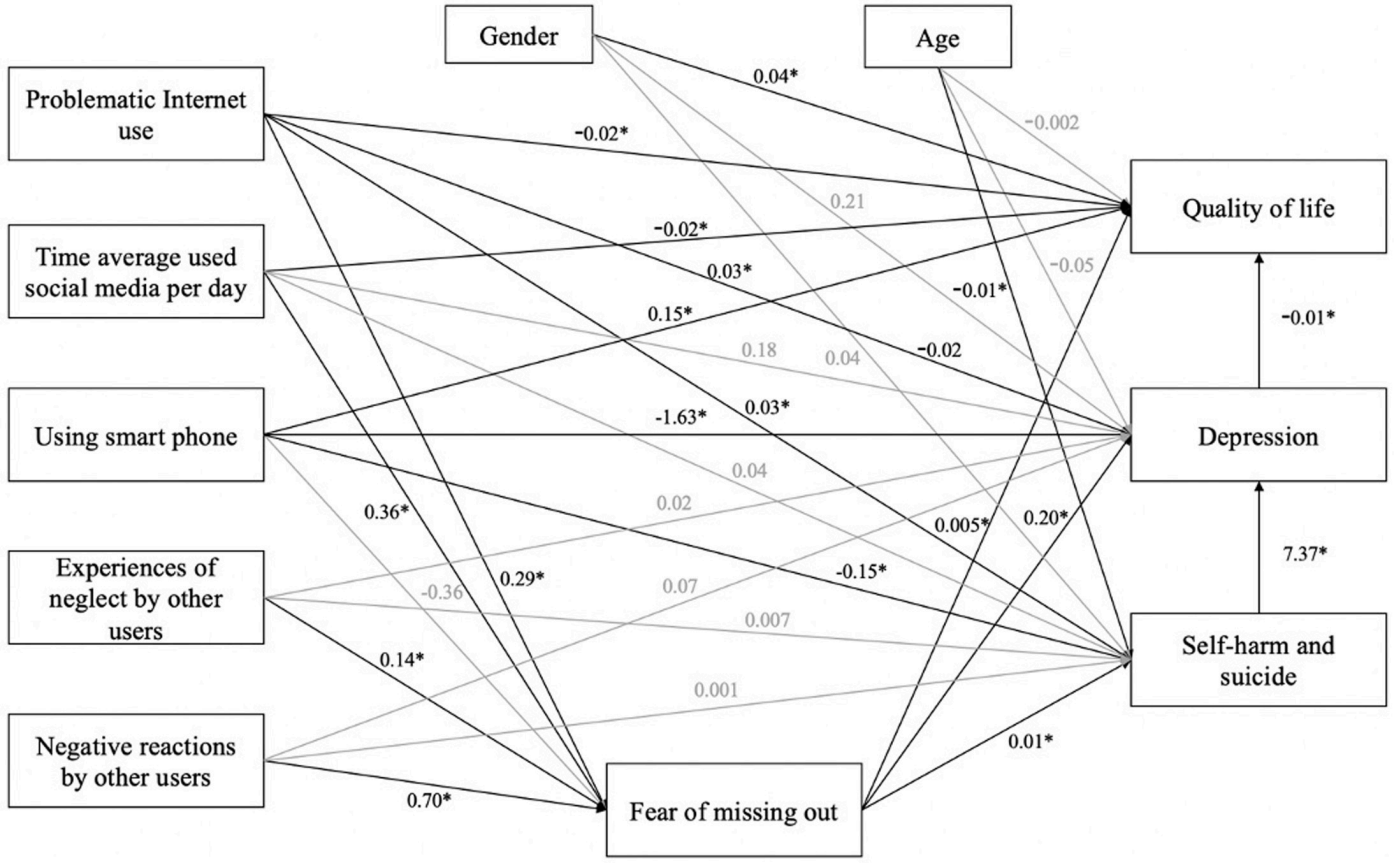
Path coefficients and p-value of structure equal model analysis.

The path coefficients and p-value of structure equal model analysis are presented in **Fig 1**. The results indicated that this structure was a good fit for RMSEA (0.057), CFI (0.988), and SRMR (0.014). Factors like “PHQ-9 score”, “Problematic Internet use”, and “Time average used social media per day”, were negatively associated with the EQ5D5L Index. By contrast, “Gender”, and “Using smartphone” were positive factors of the EQ5D5L Index. “Fear of missing out” and “self-harm and suicide” were positive factors of the PHQ-9 score while “Using smartphone” was negative. In terms of self-harm and suicide, “Fear of missing out” and “Problematic Internet use” were positive factors, by contrast, “Using smartphone” was a negative factor. The full model with structural and measurement components was presented in **S1 Appendix.**

Table 4 presented the direct and total effects.

**Table 4:** Indirect effect for relationship between some characteristics and quality of life as well as depression and self-harm and suicide by FOMO.

Pathways	Indirect effect	95% Confident interval	Total effect/ % Total effect	Direct effect/ % Direct effect
Problematic Internet use / FOMO / Quality of life	0.016*	0.008; 0.025	0.291/5.6%	0.307/5.3%
Problematic Internet use / FOMO / Depression	0.037*	0.025; 0.049	0.026; 141.7%	0.011; 339.5%
Problematic Internet use / FOMO / Self-harm and suicide	0.025*	0.015; 0.035	0.304/8.3%	0.279/9.1%
Time average used social media per day / FOMO / Quality of life	0.010*	0.004; 0.016	0.038/25.3%	0.028/33.9%
Time average used social media per day / FOMO / Depression	0.022*	0.012; 0.032	0.094/23.2%	0.072/30.2%
Time average used social media per day / FOMO / Self-harm and suicide	0.015*	0.007; 0.023	0.032/46.7%	0.017/87.6%
Using smart phone / FOMO / Quality of life	-0.001	-0.006; 0.003	0.064/2.1%	0.065/2.1%
Using smart phone / FOMO / Depression	-0.003	-0.013; 0.006	0.038/8.1%	0.035/8.8%
Using smart phone / FOMO / Self-harm and suicide	-0.002	-0.009; 0.004	0.046/4.5%	0.044/4.7%
Experiences of neglect by other users / FOMO / Depression	0.019*	0.003; 0.034	0.033; 57.1%	0.014; 133.2%
Negative reactions by other users / FOMO / Depression	0.087*	0.067; 0.107	0.132/65.8%	0.045/192.7%
Experiences of neglect by other users / FOMO / Self-harm and suicide	0.013*	0.002; 0.024	0.080/15.9%	0.067/18.9%
Negative reactions by other users / FOMO / Self-harm and suicide	0.059*	0.039; 0.079	0.067/87.9%	0.008/729.4%

*Direct Effects*: Through FOMO, both four factors “Problematic Internet use”, “Time average used social media per day”, “Experiences of neglect by other users”, and “Negative reactions by other users” were positively associated with Quality of Life, depression, and self-harm and suicide.

*Total effects*: Time average used social media per day were positively associated with Quality of Life (25.3%), depression (23.2%), and self-harm and suicide (46.7%). Two constructs “Experiences of neglect by other users” and “Negative reactions by other users” influenced both depression and self-harm and suicide. In the first category, a significant portion of the overall effect on depression, as well as self-harm and suicide, was attributed to FOMO, with 57.1% and 15.9% respectively. With regards to the impact of "Negative reactions by other users", 65.8% and 87.9% were assigned to FOMO.

## Discussion

This study is the first study to delve into social media engagement among the Vietnamese population. More importantly, it examines the links between overall well-being, FOMO, and stress associated with negative reactions provoked by social media use. Furthermore, it investigates the impact of social media usage on one’s mental health. Our results highlighted that 56.7% of those sampled were women, and there were significant differences among the genders concerning their patterns of social media use. Men tend to spend less than 2 hours on social media, as compared to women, while women spend more than 2 hours using social media. Woman participants have had more experiences about being rejected by other users, while man participants scored higher on the problematic internet use questionnaire inventory. Women participants have had an overall higher quality of life than man participants. With regard to the correlation modeling, we found a high correlation between FOMO and the PHQ-9 score.

Our results revealed a difference between the genders pertaining to their social media use. Women tend to use social media more as compared to men. This finding is congruent with that of prior research, such as that by Apricino-Martinez et al. [34]. In their cross-sectional study that sampled Spanish students aged between 17–25 years old, they reported that women, as compared to men, were more inclined towards social media usage. Prior research, such as that by Su et al. [35], has also reported there are higher levels of Internet-gaming addiction among men, and higher levels of social media addiction among women. Various factors have accounted for the gender differences in Internet and social media addiction. Dong et al. [36] has reported that gaming-related cues tend to elicit higher levels of cravings in men, as compared to women. Games also offer an element of competition, which might be more appealing to men than women [37]. In addition, the display of aggression tends to be more socially acceptable for men, and this has to some extent, influenced the design of games, whereby game designers primarily design violent and adventurous games with men in mind [38]. Women instead have a higher tendency to be addicted to social media, as social media networks allow for them to be connected socially, and women tend to value these relationships more as compared to mans [39]. In addition, there are also differences in the usage of social media between the genders. Women tend to seek out information and use social media to facilitate interpersonal connections, whereas men tend to use social media merely for recreational activities [40]. These factors might have accounted for our results, which demonstrated there to be differences between mans’ and women’s patterns of use.

Our use of the Problematic Internet Use Inventory has revealed there to be gender differences in the scores as well, with men having a higher score on the inventory. Prior studies, such as that by Shan et al. (2021) (26), who have explored the associations between Internet addiction and gender in a sample of 3380 first-year college students have revealed that men, also had significantly higher scores on the Chinese Internet Addiction scale, as compared to women. The higher scores are indicative that men are more likely to have issues with Internet-related addiction. The possible reason might be that men tend to use the Internet for various purposes. In contrast, women tend to be more limited in their use of the Internet, in that they use it mainly for chatting, updating their personal information, sending messages, and searching for information [41].

With regards to the Fear of Missing out and Stress associated with negative reactions or neglect, we found there were no differences between the genders in terms of their FOMO scores, but there was a significant difference pertaining to the stresses pertaining to the negative reasons one received from others. Considering the path analysis, we demonstrated a correlation between FOMO and experiences with neglect by others, negative reactions by others, and overall quality of life and depressive scores. Our findings are congruent with those of prior studies, such as that by Fabris et al. [19]. In their study, they examined the relationship between FOMO and emotional symptoms, by sampling a group of 472 adolescents in Italy, who were aged between 11–19. They reported that FOMO is associated with increased sensitivity to stress and increased experiences of neglect and negative reactions by others online. The authors have also reported how higher levels of FOMO are in turn associated with a decrease in well-being in individuals, which is also evident from our results, as direct path relationships were found for FOMO and quality of life, depressive scores, and risk of self-harm/suicide. One of the reasons why individuals with high levels of FOMO might be more sensitive to rejection or neglect might be because they have a higher need for group membership, and thus experienced higher levels of distress when rejected [19]. Our results about the consequential impact of FOMO are in-line with other studies. Gupta et al. [42] reported how FOMO is related to increased sleep disturbances, social anxiety, clinical depression, and a decline in one’s academic performance.

There are several clinical and research implications arising from this study. In terms of clinical implications, it is important for clinicians to that different genders might differ in their presentation regarding excessive Internet use and social media use. The fact that excessive social media use might be associated with consequential impairments in terms of quality of life and increased depressive symptoms signifies there is a need to determine if one is experiencing any other psychiatric symptoms due to one’s excessive social media use usage. Given the impact associated with excessive social media use, it is the important that individuals self-monitor their usage; and for their loved ones and caregivers to be cognizant of the potential tell-tale signs that one is using social media maladaptively, and refer the individual for early assessment and intervention. Regarding research implications, in knowing the risk factors that could predispose and continue to precipitate social media addiction, it is important to carefully examine the effectiveness of various interventional strategies. One recent approach was that of FOMO reduction (FoMo-R), which involves educating individuals about FoMO and teaching them skillsets in dealing with FoMo. This approach has been tested among 30 participants, with there being initial findings that it is effective. More work could be done in investigating such strategies. Although we have demonstrated the associations between social media and factors like FOMO, there remains a need to explore if there are any other variables that might moderate and affect the overall severity of one’s social media usage.

There are several strengths of this study. We have managed to research into a topic that is at present, not investigated. We managed to make use of online sampling and have managed to recruit a sizable group of participants. We also demonstrated the relationship between FOMO and stresses associated with neglect and rejection. Despite these strengths, there are several limitations. Most of the questionnaires that we have utilized are based on self-reported information and thus subjected to recall biases. The online sampling method might have self-selected for some individuals, and thus, the resultant sample might not be entirely representative of the Vietnamese population.

## Conclusions

This is the first study to examine social media addiction among Vietnamese adolescents, its relationship with FOMO, stresses associated with rejection and neglect, and the overall quality of life. Our results highlighted there being a relationship between FOMO and impaired overall quality of life, increased depressive symptoms, and an association between stresses relating to negative rejection and FOMO.

## Supporting information

S1 Appendix Full models including the outcome and both tier-1 and tier-2 predictors. (DOCX)

## Abbreviations

EQ-5D-5L: EuroQol-5 Dimensions-5 Levels
SS neglect: Experiences of neglect by other users
FOMO: Fear of Missing out
PHQ-9: Patient Health Questionnaire 9 items
PIUQ SF-6: Problematic Internet Use Questionnaire SF-6
SS negative: Negative reactions by other users
SS scale: Stress associated with neglect and negative reactions by online peer
