# Supplementary material for: Quality of life and mental health of adolescents: Relationships with social media addiction, Fear of Missing out, and stress associated with neglect and negative reactions by online peers
Source: PLoS One. 2023 Jun 7;18(6):e0286766. doi: 10.1371/journal.pone.0286766 (PMC10246797; doi:10.1371/journal.pone.0286766)
Supplement: S1 Appendix — (DOCX) [file pone.0286766.s001.docx]

**APPENDIX**

**Appendix 1: Full models including the outcome and both tier-1 and tier-2 predictors**

| **Structural component** | | |
| --- | --- | --- |
|  |  | **Standardised path coefficient (p-value of unstandardised estimate)** |
| **Direct paths-tier 1 to the outcome** | Fear of missing out → EQ5D5L Index | 0.005 (<0.001) |
|  | Fear of missing out → PHQ-9 score | 0.195 (<0.001) |
|  | Fear of missing out → Self-harm and suicide | 0.010 (<0.001) |
| **Direct paths-tier 2 to tier 1** | Problematic Internet use → Fear of missing out | 0.294 (<0.001) |
|  | Time average used social media per day → Fear of missing out | 0.356 (<0.001) |
|  | Using smartphone → Fear of missing out | -0.357 (0.777) |
|  | SS neglect → Fear of missing out | 0.144 (0.016) |
|  | SS negative → Fear of missing out | 0.702 (<0.001) |
| **Direct paths-tier 2 to the outcome** | Problematic Internet use → EQ5D5L Index | -0.022 (<0.001) |
|  | Time average used social media per day → EQ5D5L Index | -0.019 (0.002) |
|  | Using smart phone → EQ5D5L Index | 0.152 (0.003) |
|  | Gender → EQ5D5L Index | 0.042 (0.002) |
|  | Age → EQ5D5L Index | -0.002 (0.489) |
|  | Problematic Internet use → PHQ-9 score | -0.018 (0.531) |
|  | Time average used social media per day → PHQ-9 score | 0.183 (0.053) |
|  | Using smart phone → PHQ-9 score | -1.632 (0.046) |
|  | SS neglect → PHQ-9 score | 0.024 (0.544) |
|  | SS negative →PHQ-9 score | 0.069 (0.082) |
|  | Gender → PHQ-9 score | 0.208 (0.338) |
|  | Age → PHQ-9 score | -0.046 (0.212) |
|  | Problematic Internet use → Self-harm and suicide | 0.031 (<0.001) |
|  | Time average used social media per day → Self-harm and suicide | 0.004 (0.634) |
|  | Using smartphone → Self-harm and suicide | -0.153 (0.046) |
|  | SS neglect → Self-harm and suicide | 0.007 (0.055) |
|  | SS negative →Self-harm and suicide | 0.001 (0.780) |
|  | Gender → Self-harm and suicide | 0.036 (0.073) |
|  | Age → Self-harm and suicide | -0.007 (0.052) |
| **Direct paths outcome to the outcome** | PHQ-9 score → EQ5D5L Index | -0.012 (<0.001) |
|  | Self-harm and suicide → PHQ-9 score | 7.374 (<0.001) |
| **Goodness of fit** | RMSEA (90% CI) | 0.057 (0.040; 0.075) |
|  | CFI | 0.988 |
|  | SRMR | 0.014 |
| CFI: comparative fit index; CI: confidence interval; RMSEA: root mean square error of approximation; SRMR: Standardised root mean square residual | | |
